# Neue klinische Anwendungsbereiche der Niederfeld-Magnetresonanztomographie: Technische und physikalische Aspekte

**DOI:** 10.1007/s00117-022-00967-y

**Published:** 2022-02-22

**Authors:** Hanns-Christian Breit, Jan Vosshenrich, Michael Bach, Elmar M. Merkle

**Affiliations:** Klinik für Radiologie und Nuklearmedizin, Universitätsspital Basel, Petersgraben 4, 4031 Basel, Schweiz

**Keywords:** Bildakquisition, Signal-zu-Rausch-Verhältnis, Metallartefakte, Suszeptibilitätsartefakte, Diagnostische Qualität, Imaging acquisition, Signal-to-noise ratio, Metal artifacts, Susceptibility artifact, Diagnostic quality

## Abstract

**Hintergrund:**

Die Niederfeld-Magnetresonanztomographie (MRT) erlebt aufgrund technischer Neuerungen eine Renaissance. Die Geräte der neuen Generation bieten neue Anwendungsspektren in der Bildgebung und eine mögliche Antwort auf den steigenden Kostendruck im Gesundheitssystem.

**Fragestellung:**

Einfluss der Feldstärke auf die Technik, Physik, Bildakquisition und die diagnostische Qualität der Untersuchungen.

**Material und Methode:**

Rekapitulation der wichtigen grundlegenden physikalischen Parameter für Bildgewinnung und Qualität. Erste klinische Erfahrungen mit einem neuen 0,55-T-Niederfeldscanner.

**Ergebnisse:**

Niedrigere Feldstärken als die klinisch aktuell verbreiteten 1,5 T und 3 T sind in der Bildgewinnung durch ein zu erwartendes geringeres Signal-zu-Rausch Verhältnis gekennzeichnet. Ob dies eine diagnostische Limitation ist, muss in Studien evaluiert werden, da es verschiedene Optionen gibt, dieses vermeintliche Defizit zu kompensieren. Dies kann durch eine Verlängerung der Akquisitionszeit oder durch Einsatz von Nachverarbeitungsverfahren mit Hilfe der künstlichen Intelligenz (KI) geschehen. Zudem ist zu validieren, in welchen Körperregionen und bei welchen Krankheitsbildern die Bildqualität diagnostisch ausreichend ist. Erste Untersuchungen in unserer Klinik sind vielversprechend und zeigen beispielsweise diagnostische Qualität ohne relevanten Zeitverlust für Untersuchungen der Lendenwirbelsäule. Potenzielle Stärken aufgrund geringerer Suszeptibilitätsartefakte ergeben sich in der Lungenbildgebung oder bei Implantaten.

**Schlussfolgerung:**

Niederfeldscanner bieten eine Vielzahl von neuen Anwendungsfeldern mit feldstärkebedingten Vorteilen. Bei den meisten anderen klinischen Untersuchungsfeldern kann mindestens eine diagnostische Qualität erwartet werden.

## Abstract (en)

**Background:**

Low-field magnetic resonance imaging (MRI) is experiencing a renaissance due to technical innovations. The new-generation devices offer new applications for imaging and a possible solution to increasing cost pressures in the healthcare system.

**Objectives:**

Effects of field strength on technique, physics, image acquisition, and diagnostic quality of examinations are presented.

**Methods:**

Important basic physical parameters for image acquisition and quality are summarized. Initial clinical experience with a new 0.55 T low-field scanner is presented.

**Results:**

Field strengths that are lower than the currently used 1.5 T and 3 T field strengths are characterized by an expected lower signal-to-noise ratio in image acquisition. Whether this is a diagnostic limitation needs to be evaluated in studies, as there are several options to offset this perceived drawback, including increasing measurement time or artificial intelligence (AI) postprocessing techniques. In addition, it is necessary to meticulously investigate whether low-field systems allow diagnostically adequate image quality to be achieved in different body regions and different disease entities. Initial studies in our clinic are promising and show, for example, diagnostic quality without relevant loss of time for examinations of the lumbar spine. Advantages of low-field MRI include reduced susceptibility artifacts when imaging the lungs and in patients with metallic implants.

**Conclusion:**

Low-field scanners offer a variety of new fields of application with field strength-related advantages. In most other clinical examination fields, at least diagnostic quality can be expected.

Die Magnetresonanztomographie (MRT) ist ein elementarer Baustein in der medizinischen Diagnostik sowohl für internistische, neurologische, chirurgische als auch orthopädische Fragestellungen und heutzutage nicht mehr aus Klinik und ambulanter Praxis wegzudenken. Es dominieren Scanner mit Magnetfeldstärken von 1,5 T und 3 T. Aufgrund verschiedener technischer Innovationen ergeben sich jedoch neue Chancen für Scanner mit niedrigeren Feldstärken. Ziel dieser Arbeit ist es, die physikalischen Einflüsse der Feldstärke auf die klinische Bildgebung zu beleuchten.

Die MRT-basierte Bildgebung ist eine Erfolgsgeschichte der Radiologie und der diagnostischen Medizin. Von den Anfängen in den 1980er Jahren mit einigen hundert Geräten ist die MR-basierte Bildgebung mittlerweile ebenso wie die Computertomographie (CT) in der westlichen Welt nahezu überall in der Routine verfügbar [15]. Weltweit waren 2018 mehr als 36.000 Scanner im Einsatz bei einem jährlich zu erwartenden Zuwachs von 2500 Geräten, wobei nach wie vor ausgeprägte regionale Unterschiede existieren [12]. Dabei dominieren Geräte mit Feldstärken von 1,5 T bis 3 T, während Ultrahochfeld-MRT mit 7 T aktuell wenig verbreitet sind.

MR-Systeme mit Niederfeldstärke operieren in einem Bereich von 0,35 T bis 0,6 T und erleben nach ersten klinischen Einsätzen in der Anfangszeit der MRT-Bildgebung aktuell eine Renaissance [14]. Erfahrungen über das Anwendungsspektrum von Niederfeldstärke-MRT im Vergleich zu Geräten höherer Feldstärke stammen überwiegend aus den 1980er und 1990er Jahren. Aufgrund technischer Entwicklungen im Bereich des Spulen- und Gradientenbaus, aber auch im Bereich der Bildnachbearbeitung eröffnete sich in den letzten Jahren ein neuer Blickwinkel auf die Niederfeldtechnik mit neuen Anwendungsmöglichkeiten im Bereich der interventionellen Radiologie [1], der Lungenbildgebung [2, 5] oder auch der muskuloskeletalen Bildgebung [4].

Vorteile dieser Niederfeldgeräte sind sowohl geringere Kosten in der Herstellung von Magneten, Gradienten und Spulen als auch geringere bauliche Anforderungen durch ein deutlich niedrigeres Gewicht und weniger Anforderungen an die Abschirmung in einem Faradaykäfig. Weitere Vorteile sind eine mögliche Reduzierung des durch die Gradienten erzeugten Schalllevels und eine daraus resultierende Steigerung des Patientenkomforts, der zu einer noch höheren Akzeptanz der Patienten führen kann. Klinisch relevant ist dies zudem bei der Untersuchung von Schwangeren oder pädiatrischen Patienten durch ein niedrigeres Stresslevel während der Untersuchung [13]. Zudem führt die niedrigere Feldstärke zu einer Erhöhung der gefühlten und faktischen Patientensicherheit. So korrelieren die physikalischen Kräfte und daraus resultierenden Gefahren metallischer Fremdkörper mit der Feldstärke. Auch etwaige Gefahren wie beispielsweise Hitzeentwicklung durch Implantate, Prothesen oder Tätowierungen sollten bei niedrigeren Feldstärken geringer sein.

Eine weitere Entwicklungsperspektive ist die Verwendung größerer Bohrungen mit einem Zugewinn an Patientenkomfort und der Möglichkeit einer einfachen Patientenüberwachung sowie ggf. der Verzicht auf Sedationen oder Narkosen bei klaustrophobischen Patienten. Zudem bieten sich durch die bessere Zugänglichkeit des Patienten neue Möglichkeiten in der interventionellen Radiologie an [7].

Ziel dieses Beitrags ist es, eine Übersicht über die physikalischen Grundlagen der Bildgebung in Abhängigkeit von der Feldstärke zu liefern und dabei die Vor- und Nachteile, die sich daraus ergeben, zu diskutieren.

## Physikalische Aspekte der Bildgebung

### Technische Voraussetzungen

Im Bereich der Niederfeld-MRT ist im Gegensatz zu kommerziellen 1,5 T und 3 T eine Erzeugung des Magnetfelds sowohl mit Permanentmagneten, konventionellen Elektromagneten als auch mit supraleitenden Magneten möglich. Gleichzeitig ist der Magnet der Hauptkostenpunkt eines MRT-Systems, Preis und Feldstärke korrelieren miteinander linear.

### Kontrast und Signal-zu-Rausch-Verhältnis in Abhängigkeit der Feldstärke

Eines der Hauptargumente für höhere Feldstärken war und ist das bessere Signal-zu-Rausch-Verhältnis (SNR). Das Signal einer MR-Messung ist zum einen proportional zu der erreichten Magnetisierung, welche ihrerseits proportional zur Magnetfeldstärke (B) ist. Zum anderen ist die in den Empfangsspulen induzierte Spannung proportional zur Lamorfrequenz, welche ebenfalls proportional zur Magnetfeldstärke ist. Das MR-Signal hängt somit quadratisch von der Magnetfeldstärke ab. Gleichzeitig ist das Rauschen in dem Bereich der hier betrachteten Magnetfeldstärke in etwa proportional zu B^1/2. Das SNR hängt somit in guter Näherung von B^3/2 ab. Aufgrund der mit der Feldstärke abnehmende Fett-Wasser-Verschiebung kann die Datenaufnahme bei niedrigeren Feldstärken mit geringeren Bandbreiten erfolgen, was zu einem Signalgewinn führt.

**Figure 1:**
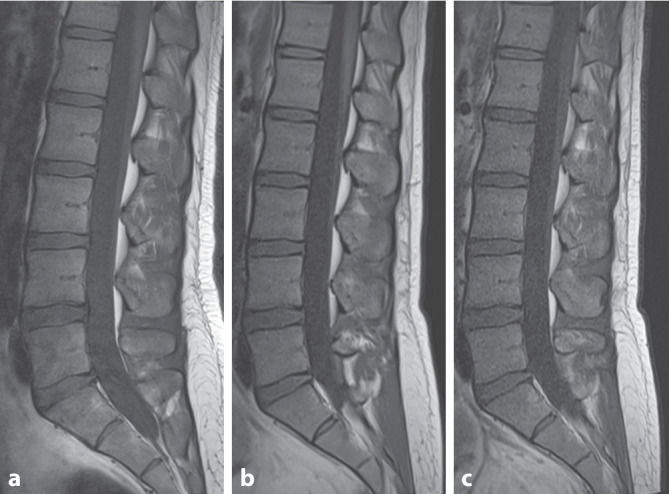

Neue Nachbearbeitungsmethoden, die auf künstlicher Intelligenz (KI) basieren, können die sichtbare Auflösung erhöhen, das Bildrauschen unterdrücken oder zu einer Messzeitverkürzung genutzt werden (Abb. 1; Tab. 1; [8, 10, 11]).

**Table 1:** 

	T1-TSE sagittal			T2-TSE sagittal		
	1,5 T	0,55 T	0,55 T*	1,5 T	0,55 T	0,55 T*
TR (ms)	625	454	454	3600	3500	3500
TE (ms)	11	13	13	102	99	96
ST (mm)	4	4	4	4	4	4
Auflösung (mm^2^)	0,7 × 0,7	0,8 × 0,8	0,5 × 0,5	0,7 × 0,7	0,8 × 0,8	0,5 × 0,5
FOV (mm^2^)	300 × 300	320 × 320	320 × 320	300 × 300	320 × 320	320 × 320
TA (min)	02:29	05:26	02:28	01:44	03:34	03:23

Eine sehr effektive Methode, um das SNR zu erhöhen, ist eine moderate Reduktion der Auflösung. Bei einer Verringerung der Auflösung von 1 mm auf 1,1 mm isotrop beträgt der Signalgewinn 30 %. Tatsächlich kann der Signalverlust durch die geringere Feldstärke bei 0,55 T gegenüber 1,5 T so hauptsächlich durch eine leichte Reduktion der Auflösung und moderate Verlängerung der Messzeit kompensiert werden. Exemplarisch finden sich auf 0,55 T adaptierte Sequenzen der Lendenwirbelsäule und des Neurokraniums im Vergleich zu 1,5 T in den Tab. 1 und 2.

**Table 2:** 

	DWI		FLAIR		SWI	
	1,5 T	0,55 T	1,5 T	0,55 T	1,5 T	0,55 T
TR (ms)	6200	7400	8510	7780	49	172
TI (ms)	–	–	2120	2369	–	–
TE (ms)	103	102	112	96	40	100
ST (mm)	3	3	3	3	3	3
Auflösung (mm^2^)	1,44 × 1,44	1,67 × 1,67	0,9 × 0,9	1,28 × 1,03	0,94 × 0,8	1,12 × 0,9
FOV (mm^2^)	230 × 230	220 × 220	187 × 230	209 × 230	201 × 230	214 × 288
TA (min)	02:04	04:35	01:44	04:56	01:52	02:37

Für den Bildeindruck sind der Kontrast und das Kontrast-zu-Rausch-Verhältnis entscheidend [6]. In der Literatur gibt es kaum aktuelle oder eindeutige Studien bezüglich der diagnostischen Performance in Abhängigkeit von der Feldstärke und somit kaum Evidenz hinsichtlich des diagnostischen Zugewinns bei höheren Feldstärken. Es existieren zwar zahlreiche Studien, die belegen, dass bei höheren Feldstärken die menschliche Anatomie mit einem höheren Detailgrad abgebildet und kleinere anatomische Strukturen abgegrenzt werden können. Ob dies jedoch einen entscheidenden Effekt auf die Diagnose oder gar Therapie, Morbidität und Mortalität des Patienten hat, wird dabei meistens nicht analysiert.

**Figure 2:**
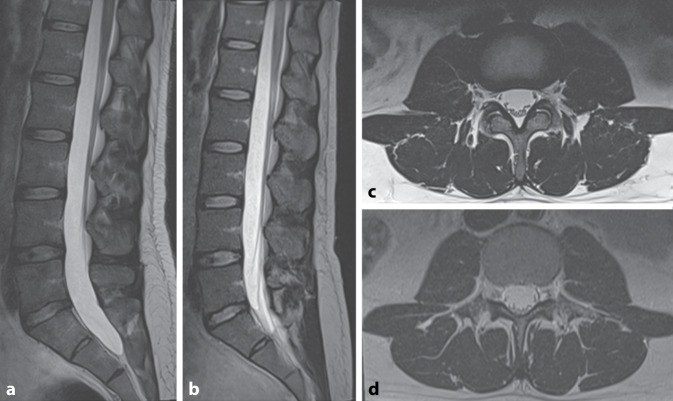

Die Datenlage ist insgesamt widersprüchlich und selbst alte Studien mit Scannern der ersten Generation zeigten zum Teil keinen diagnostischen Zugewinn bei höheren Feldstärken [10, 17]. In ersten Untersuchungen am Universitätsspital Basel wurden beispielsweise keine Einschränkungen im Hinblick auf die diagnostische Aussagekraft bei Wirbelsäulenuntersuchungen an dem 0.55T Siemens Magnetom Free.Max (Siemens Healthineers, Erlangen, Deutschland) verglichen mit einem 1.5T Siemens Magnetom Avanto (Siemens Healthineers, Erlangen, Deutschland) der klinischen Routine gefunden (siehe Abb. 2).

### T_1_- und T_2_-Zeiten

Die longitudinale Relaxationszeit T_1_ ist abhängig von der Feldstärke und dem in der Bildgebung dargestellten Organ und seiner Umgebung [9]. Dabei korrelieren Feldstärke und T_1_-Zeit in menschlichem Gewebe positiv. Die verschiedenen Organe und Bestandteile des menschlichen Körpers haben bei 0,55 T also kürzere T_1_-Zeiten als bei 1,5 T und 3 T. Daher sind prinzipiell für T1-gewichtete Sequenzen bei 0,55 T kürzere Repetitionszeiten und damit Akquisitionszeiten möglich als bei höheren Feldstärken. Dieser Effekt wird jedoch durch das geringere Signal und die damit verbundene Notwendigkeit zur Akquisition mehrerer Mittelungen zum Teil konterkariert.

Die transversale Relaxationszeit T_2_ ist in der Theorie relativ unabhängig von der Feldstärke [16]. Daher ist zu erwarten, dass Suszeptibilitätseffekte bei niedrigeren Feldstärken geringer ausfallen und so bei einem längeren T_2_* der Signalabfall beispielsweise durch verschiedene metallische Fremdkörper, Gas oder Luft weniger stark ist. Campbell-Washburn et al. zeigten in einer Untersuchung an 83 Patienten mit einem experimentellen 0,55-T-Scanner, dass die T_1_-Zeiten verschiedener Gewebe durchschnittlich 32 % kürzer sind als bei 1,5 T. Dahingegen wurden durchschnittlich 26 % längere T_2_- und 40 % längere T_2_*-Zeiten beobachtet [1].

### Artefakte

Die Artefaktreduktion, insbesondere bei metallischen Fremdmaterialien, ist einer der großen Vorteile von Niederfeldgeräten. Dies ist insbesondere aufgrund einer älter werdenden Bevölkerung mit einem zu erwartenden deutlichen Anstieg an Fremdmaterial, wie beispielsweise Hüftprothesen und Herzschrittmachern von großer Bedeutung. Die Ausdehnung der durch Implantate verursachten Artefakte lässt sich anhand der folgenden Gleichung abschätzen:

$$\text{Artefaktausdehnung }\propto (\Updelta \text{ Suszeptibilit"at *}\mathrm{B}\text{* TE})/\text{ Bandbreite}$$

**Figure 3:**
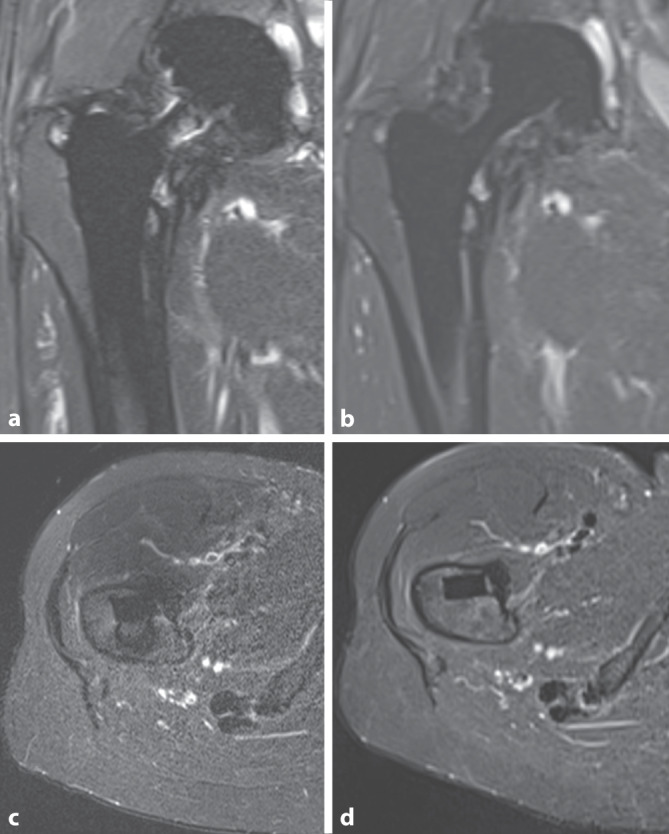

Erste eigene klinische Erfahrungen zeigen ebenfalls eine Artefaktreduktion eines 0,55 T Siemens Magnetom Free.Max verglichen mit einem 1,5 T Magnetom Avanto, wie in Abb. 3 am Beispiel eines Patienten mit einer Hüftgelenkendoprothese zu sehen ist.

Die geringeren Suszeptibilitätseffekte bei niedrigen Feldstärken sind auch sehr günstig für die Bildgebung der Lunge, und es konnten vielversprechende Ergebnisse dabei erzielt werden [2].

### Chemical Shift

Chemical shift beschreibt im Wesentlichen die Abhängigkeit der Larmorfrequenz eines Protons von seiner chemischen Umgebung. So weisen beispielsweise Wasserstoffprotonen in Wasser eine gering unterschiedliche Larmorfrequenz auf als in Fett. Dieser Effekt korreliert proportional mit der Feldstärke B; für den Frequenzunterschied gilt

$$\Updelta f=147\mathrm{\,Hz}/\mathrm{T}\mathrm{*}\mathrm{B}.$$

Dies hat zur Folge, dass der Frequenzunterschied bei 0,55 T um etwa ein Drittel niedriger ist als bei 1,5 T. Durch den geringeren Frequenzunterschied sind spektrale Methoden der Fettsättigung im Hinblick auf die Homogenität bei niedrigeren Feldstärken anspruchsvoller. Dies liegt daran, dass ein spektraler Sättigungspuls ein noch geringeres Frequenzspektrum abdecken darf, um gezielt ausschließlich Fettgewebe zu unterdrücken.

Auswirkungen hat dies auch auf die zunehmend an Bedeutung gewinnende DIXON-Technologie [3]. Bei dieser Technik werden Bilder mit Echozeiten generiert, bei denen sich wasser- und fettgebundene Wasserstoffprotonen in gleicher („in phase“) oder entgegengesetzter Phase („opposed phase“) befinden. Basierend auf diesen Bilddaten, können fett- oder wassersupprimierte Bilder erzeugt werden, so dass durch eine Sequenz effektiv 4 Kontraste generiert werden [11]. Techniken, die noch weitere Echos generieren, ermöglichen die quantitative Bestimmung des Fett- oder Eisengehalts verschiedener Gewebe. Durch den geringeren Frequenzunterschied bei 0,55 T sind längere Echozeiten erforderlich und der Signalabfall zum Zeitpunkt des Echos entsprechend höher. Dies resultiert in einem schlechterem Signal-zu-Rausch Verhältnis der akquirierten Bilder.

## Zukunft

Niederfeld-MRT-Geräte versprechen zum einen aufgrund der zu erwartenden geringeren Suszeptibilitätsartefakte einen vielversprechenden Anwendungsbereich im Bereich der Bildgebung von Endoprothesen, bei Patienten mit Implantaten, wie Schrittmachern oder Portsystemen und auch bei der Untersuchung der Lunge. Dabei könnte Letzteres als strahlenfreie Alternative zu Röntgen- oder CT-Aufnahmen, insbesondere bei Kindern zur Beurteilung von Infekten oder aber auch mediastinalen Raumforderungen, dienen. Im Hinblick auf Signal und Auflösung sind zweifelsohne Abstriche gegenüber 1,5-T- und 3‑T-Scannern zu machen. Weitere Studien müssen klären, welche Anforderungen hinsichtlich Bildqualität und Patientenbelastung bei welchen klinischen Anforderungen erfüllt sein müssen, um zu einem optimalen Outcome zu kommen.

Für Niederfeldgeräte bietet sich durch die niedrigeren Anschaffungs- und Unterhaltskosten sowie geringere bauliche Anforderungen an den Scannerraum zudem ein erweitertes Spektrum für die Verwendung der MRT-Bildgebung an. Beispielsweise könnten Systeme näher an Intensivstationen oder Notaufnahmen platziert werden und so eine schnellere Verfügbarkeit der MRT-Bildgebung für schwerkranke Patienten ermöglichen. Auch ein Einsatz in „Low-income“-Ländern könnte eine bisher bestehende Versorgungslücke schließen. Limitationen aufgrund der niedrigeren Feldstärke sind dabei für die meisten Anwendungsfelder nicht zu erwarten.

## Fazit für die Praxis

- Durch technische Neuerungen erleben MRT-Scanner mit niedrigen Feldstärken um 0,55 T eine Renaissance.
- Physikalisch bedingte Nachteile, wie ein geringeres Signal im Vergleich zu Scannern mit 1,5 T und 3 T können durch Fortschritte der Gradiententechnologie, der parallelen Bildgebung und vielfältige neue Möglichkeiten der Bildnachverarbeitung kompensiert werden.
- Geringere Suszeptibilitätsartefakte versprechen bessere Resultate in der Lungenbildgebung und bei Patienten mit Implantaten oder anderen Fremdkörpern.
- Weitere Vorteile sind die deutlich geringeren Anschaffungs‑, Installations- und Unterhaltskosten.
- Eine sorgfältige Evaluation der Bildqualität im Hinblick auf etwaige Einschränkungen in der Diagnostik ist dabei für jede Fragestellung notwendig.
